# Correction to ‘Different epitopes of *Ralstonia solanacearum* effector RipAW are recognized by two *Nicotiana* species and trigger immune responses’

**DOI:** 10.1111/mpp.13420

**Published:** 2024-01-19

Niu Y, Fu S, Chen G, Wang H, Wang Y, Hu J, Jin X, Zhang M, Lu M, He Y, Wang D, Chen Y, Zhang Y, Coll N, Valls M, Zhao C, Chen Q and Lu H. Different epitopes of *Ralstonia solanacearum* effector RipAW are recognized by two *Nicotiana* species and trigger immune responses. *Molecular Plant Pathology* 2022;23:188–203

In the authorship section, the affiliations ‘Dongdong Wang^2^, Yong Zhang^3,4^, Núria S. Coll^5^, Marc Valls^5,6^, Qin Chen^2^ and Haibin Lu^1,7^’ (Author's affiliations) were incorrect. They should have read ‘Dongdong Wang^3^, Yong Zhang^4,5^, Núria S. Coll^6^, Marc Valls^6,7^, Qin Chen^3^ and Haibin Lu^1,2^’

We apologize for these errors.

